# Risk Factors of Atrial Arrhythmia in Patients With Liver Cirrhosis: A Retrospective Study

**DOI:** 10.3389/fcvm.2021.704073

**Published:** 2021-07-05

**Authors:** Xiya Lu, Zhijing Wang, Liu Yang, Changqing Yang, Meiyi Song

**Affiliations:** Division of Gastroenterology and Hepatology, Digestive Disease Institute, Shanghai Tongji Hospital, Tongji University School of Medicine, Shanghai, China

**Keywords:** liver cirrhosis, atrial arrhythmia, age, ascites, risk factor

## Abstract

**Background and Objectives:** Liver cirrhosis is known to be associated with atrial arrhythmia. However, the risk factors for atrial arrhythmia in patients with liver cirrhosis remain unclear. This retrospective study aimed to investigate the risk factors for atrial arrhythmia in patients with liver cirrhosis.

**Methods:** In the present study, we collected data from 135 patients with liver cirrhosis who were admitted to the Department of Gastroenterology at Shanghai Tongji Hospital. We examined the clinical information recorded, with the aim of identifying the risk factors for atrial arrhythmia in patients with liver cirrhosis. Multiple logistic regression analysis was used to screen for significant factors differentiating liver cirrhosis patients with atrial arrhythmia from those without atrial arrhythmia.

**Results:** The data showed that there were seven significantly different factors that distinguished the group with atrial arrhythmia from the group without atrial arrhythmia. The seven factors were age, white blood cell count (WBC), albumin (ALB), serum Na^+^, B-type natriuretic peptide (BNP), ascites, and Child-Pugh score. The results of multivariate logistic regression analysis suggested that age (β = 0.094, OR = 1.098, 95% CI 1.039–1.161, *P* = 0.001) and ascites (β =1.354, OR = 3.874, 95% CI 1.202–12.483, *P* = 0.023) were significantly associated with atrial arrhythmia.

**Conclusion:** In the present study, age and ascites were confirmed to be risk factors associated with atrial arrhythmia in patients with liver cirrhosis.

## Introduction

The prevalence of liver cirrhosis caused by alcohol consumption, as well as viral, immune, and other potentially pathogenic factors are expected to increase in the coming decades. The interaction between the liver and heart has been described previously (1, 2). First, the heart and liver are common target organs of pathogenic factors, such as alcohol consumption and infection (3, 4). Cardiac complications are not rare during decompensated liver cirrhosis, including diastolic and systolic dysfunction and cardiac electrophysiological remodeling, known as cirrhotic cardiomyopathy (5). Cardiac complications of liver cirrhosis are typically the result of medications, hemodynamic disorders, infections, inflammatory states, and other unknown factors.

As previously reported, patients with liver cirrhosis have an increased risk of atrial arrhythmia even without underlying heart disease (6, 7). This risk does not decrease following liver transplantation. Atrial arrhythmia has been associated with a higher risk of perioperative cardiovascular events and poorer long-term prognoses (8). Atrial fibrillation or flutter, atrial premature beat, and atrial tachycardia are common atrial arrhythmias. Anticoagulation treatment is an important therapeutic element that is used to prevent stroke in patients with atrial fibrillation. However, anticoagulants significantly increase the risk of esophageal and gastric variceal bleeding in patients with liver cirrhosis, concomitantly increasing the risk of death (5, 9). The choice of anticoagulation therapy in patients with liver cirrhosis is more challenging than it is in patients without chronic liver disease (10). Age, obesity, diabetes, hypertension, and cardiovascular disease are known to increase the risk of new-onset atrial arrhythmia (11–14); however, the risk factors for atrial arrhythmia in patients with liver cirrhosis are not fully understood.

In this retrospective study, we examined the medical records of cirrhotic patients with or without atrial arrhythmia to screen for risk factors associated with atrial arrhythmia. Our study aimed to identify the risk factors for new-onset atrial arrhythmia in patients with liver cirrhosis, in order to provide a potential method for predicting atrial arrhythmia in this patient population.

## Methods

### Experiment Design and Participants

**Figure 1:**
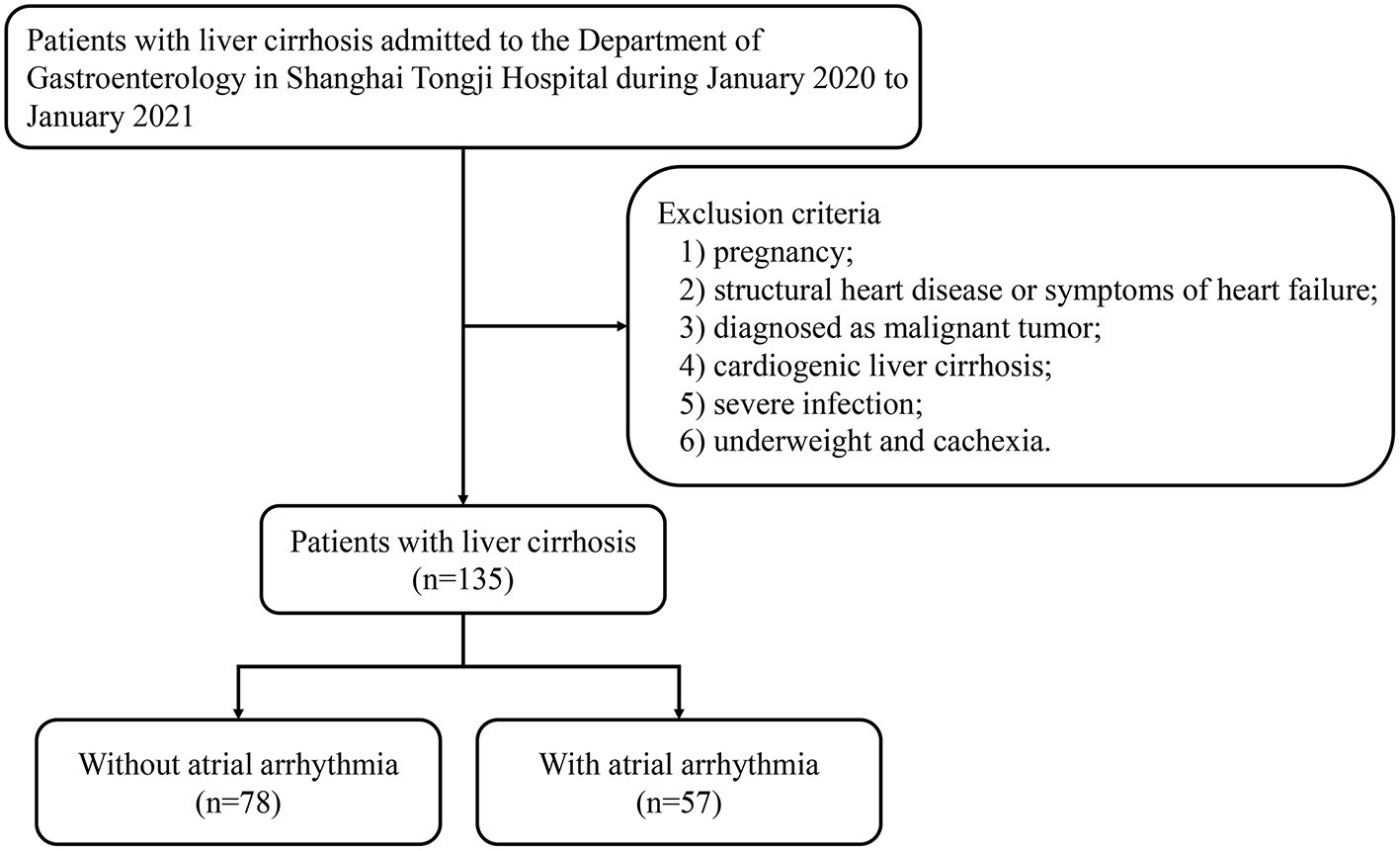
Patient flow diagram.

We enrolled patients with liver cirrhosis who were admitted to the Department of Gastroenterology at Shanghai Tongji Hospital between January 2020 and January 2021, as shown in Figure 1. All medical information was obtained from electronic medical records. The exclusion criteria were as follows: (1) pregnancy; (2) structural heart disease or symptoms of heart failure; (3) diagnosis of malignant tumor; (4) cardiogenic liver cirrhosis; (5) severe infection; and (6) underweight and cachexia. A total of 135 patients were included in the study. This study was approved by the Ethics Committee of Tongji Hospital Affiliated to Tongji University.

This study used PASS 15 to calculate the sample size. The minimal significance (α) and statistical power (1 – β) were set at 0.05 and 0.80, respectively. According to the calculation results, the minimum sample size of the control group is 55, the arrhythmia group is 74, and total sample size is 129 as shown in Table 1.

**Table 1:** Calculation of sample size using PASS 15.

**α**	**1-β**	**Control**	**Atrial arrhythmia**	**Total**
0.05	0.80	74	55	129

Ascites was diagnosed according to the guidelines of the International Ascites Club (15), as follows: mild, only detectable by ultrasound; moderate, evident by moderate symmetrical distension of the abdomen; and severe, with marked abdominal distension.

Hepatic encephalopathy was diagnosed according to AASLD/EASL guidelines (16), as follows: Grade I, mild mental and behavioral changes; Grade II, significant personality change; Grade III, lethargy; and Grade IV, coma.

Gastroesophageal varices were diagnosed by endoscopy and were divided into two subgroups: (1) no variceal bleeding during this admission and (2) with variceal bleeding during this admission.

### Clinical Information

General information, including age, sex, body weight, body mass index [BMI, weight (kg)/height (m)^∧^2], etiology of liver cirrhosis, blood pressure, pulse pressure, past medical history, and complications of liver cirrhosis were collected from the patient records. The blood pressure of patients was measured using a mercury sphygmomanometer after 5 min in a seated position. Blood pressure was determined as the average of two measurements. The etiology of liver cirrhosis was divided into five classes: alcoholic liver cirrhosis, hepatitis B liver cirrhosis, hepatitis C liver cirrhosis, immune-related liver cirrhosis, and others.

Biochemical characteristics included hemoglobin (Hb), white blood cell count (WBC), platelet count (PLT), prothrombin time (PT), aspartate aminotransferase (AST), alanine aminotransferase (ALT), glutamyltransferase (γ-GT), alkaline phosphatase (AKP), total bilirubin, direct bilirubin, albumin (ALB), serum K^+^, serum Na^+^, serum Cl^−^, creatinine, B-type natriuretic peptide (BNP), and troponin. Venous blood was collected from all patients on the morning of the first day after admission, and biochemical characteristics were detected using an automatic biochemical analyzer.

### Electrocardiogram Detection

The patients were kept in a horizontal position for 5 min to recover their resting heart rate and underwent ECG detection using a 12-lead synchronous electrocardiograph. The voltage was 10 mm/mV, and the lead wires and electrodes were connected after wiping the skin with 75% ethanol. The paper speed was set to 25 mm/s, and at least five consecutive cardiac cycles were recorded. The ECG diagnosis was performed by two experienced electrocardiologists in a double-blinded manner. A total of 135 patients included in the study were divided into two groups: those without arrhythmia and those with arrhythmia, including atrial tachycardia, atrial fibrillation, atrial flutter, and atrial premature beats.

### Portal Vein Doppler Ultrasound Detection

A TOSHIBA Apolio 500 color Doppler ultrasound diagnostic apparatus with a probe frequency of 5 MHz was used for this study. The patients were instructed to fast for 12 h before the ultrasound examination. The tilt probe was adjusted so that the angle between the Doppler sound velocity and the blood vessel path was 60°, and the inner diameter and the blood flow velocity of the main portal vein and the main splenic vein could be measured (17, 18).

### Child-Pugh Score

The Child-Pugh score was calculated using ascites, hepatic encephalopathy, total bilirubin, ALB, and PT extension, as previously described (19).

### Statistical Analysis

The measurement data are presented as mean ± SD, and the Student's *t*-test was used to compare the characteristic differences between the two groups. Categorical data are recorded as the frequency of categorical variables, and the Chi-square test was used to compare the characteristic differences between the categorical variable groups. *P*-value (*P*) < 0.05 was considered statistically significant. In the results of the Student's *t*-test and Chi-square test, factors with statistical differences were selected for multiple logistic regression analysis to calculate the odds ratio (OR) and 95% confidence interval (CI).

## Results

### Main Clinical Characteristics of Patients

The main characteristics of patients with liver cirrhosis are presented in Table 2. A total of 135 liver cirrhosis patients included [61 men (45.19%) and 74 women (54.81%)], and the age of liver cirrhosis patients was 66.56 ± 12.07 years. Furthermore, body weight of liver cirrhosis patients was 60.56 ± 8.34 kg, and BMI was 21.51 ± 2.01 kg/m^2^. Among the liver cirrhosis patients, 17 patients (12.59%) had alcohol-related liver cirrhosis, 45 patients (33.33%) had HBV-related liver cirrhosis, 13 (9.63%) had HCV-related liver cirrhosis, 31 (22.96%) had liver cirrhosis caused by autoimmune diseases, and 29 (22.96%) had liver cirrhosis related to other causes. Among 57 liver cirrhosis patients with atrial arrhythmia, there were 14 (24.56%) patients with atrial tachycardia, 4 (7.02%) patients with atrial fibrillation, 7 (12.28%) patients with atrial flutter, and 32 (56.14%) patients with atrial premature beats. Only age was a significant factor impacting atrial arrhythmia. Liver cirrhosis patients with atrial arrhythmia (73.54 ± 8.21 years) were significantly older than those without atrial arrhythmia (61.45 ± 11.92 years) (*P* < 0.001).

**Table 2:** Main characteristics of liver cirrhosis patients with or without atrial arrhythmia.

**Variable**	**Control**	**Atrial arrhythmia**	**Total**	**χ^2^**	***P***
	**(*n* = 78)**	**(*n* = 57)**	**(*n* = 135)**		
**Gender**					
Male	36	25	61 (45.19%)	0.070	0.791
Female	42	32	74 (54.81%)		
Body weight (kg)	60.86 ± 8.04	60.14 ± 8.79	60.56 ± 8.34	/	0.623
BMI (kg/m^2^)	21.49 ± 1.93	21.55 ± 2.15	21.51 ± 2.01	/	0.852
Age (years)	61.45 ± 11.92	73.54 ± 8.21	66.56 ± 12.07	/	**<0.001**
**Etiology of liver cirrhosis**					
Alcoholic	9	8	17 (12.59%)	2.295	0.682
Hepatitis B	29	16	45 (33.33%)		
Hepatitis C	6	7	13 (9.63%)		
Immune	19	12	31 (22.96%)		
Others	15	14	29 (22.96%)		
**Atrial arrhythmia**					
Atrial tachycardia	0	14 (24.56%)	/	/	/
Atrial fibrillation	0	4 (7.02%)	/		
Atrial flutter	0	7 (12.28%)	/		
Atrial premature beats	0	32 (56.14%)	/		

*The bold values of “P” represent statistical differences between control and atrial arrhythmia group*.

### Biochemical Characteristics and Portal Vein Doppler Ultrasound of Liver Cirrhosis Patients With or Without Atrial Arrhythmia

The biochemical characteristics and portal vein Doppler ultrasound results of patients with liver cirrhosis are presented in Table 3. The results showed that there were significant differences in WBC, ALB, serum Na^+^, and BNP between the two groups. However, no significant differences in the inner diameter and the blood flow velocity of the main portal vein and the main splenic vein were observed. The WBC (6.22 ± 3.25 × 10^9^/L) and BNP (295.08 ± 308.15 pg/mL) in liver cirrhosis patients with atrial arrhythmia were significantly higher than those in patients without atrial arrhythmia (WBC: 4.59 ± 2.44 × 10^9^/L, BNP: 97.14 ± 142.51 pg/mL). The ALB (6.22 ± 3.25 × 10^9^/L) and serum Na^+^ (137.22 ± 6.05 mmol/L) in liver cirrhosis patients with atrial arrhythmia were significantly lower than that in the group without atrial arrhythmia (ALB: 32.56 ± 6.91 g/L, serum Na^+^: 139.46 ± 4.65 mmol/L).

**Table 3:** The biochemical characteristics and portal vein doppler ultrasound of liver cirrhosis patients with or without atrial arrhythmia.

**Variable**	**Control**	**Atrial arrhythmia**	**Total**	***P***
	**(*n* = 78)**	**(*n* = 57)**	**(*n* = 135)**	
Systolic blood pressure (mmHg)	123.82, 13.61	123.32, 19.35	123.61, 16.22	0.866
Diastolic blood pressure (mmHg)	74.33, 8.36	72.14, 12.57	73.41, 10.36	0.255
Pulse Pressure	49.49, 13.85	51.18, 16.40	50.20, 14.94	0.519
**Laboratory findings**				
HB (g/L)	108.53, 27.38	104.44, 72.20	106.80, 51.12	0.648
WBC (×10^9^/L)	4.59, 2.44	6.22, 3.25	5.27, 2.91	**<0.001**
PLT (×10^9^/L)	84.94, 59.54	104.44, 63.18	93.17, 61.63	0.069
PT (s)	14.16, 2.56	14.97, 3.56	14.49, 3.03	0.154
AST (U/L)	66.31, 121.84	54.84, 59.33	61.47, 100.17	0.513
ALT (U/L)	41.23, 71.13	29.82, 34.41	36.41, 58.60	0.266
γ-GT (U/L)	67.42, 68.72	91.21, 141.74	76.90, 104.22	0.289
AKP (U/L)	135.93, 75.68	141.77, 130.32	138.25, 100.56	0.759
Total bilirubin (μmol/L)	44.02, 76.03	48.56, 69.86	45.94, 73.26	0.724
Direct bilirubin (μmol/L)	17.81, 36.91	19.32, 32.35	18.45, 34.94	0.806
ALB (g/L)	32.56, 6.91	28.49, 5.16	30.84, 6.53	**0.003**
Serum Na^+^ (mmol/L)	139.46, 4.65	137.22, 6.05	138.51, 5.38	**0.022**
Serum K^+^ (mmol/L)	3.74, 0.57	3.99, 0.87	3.85, 0.72	0.057
Serum Cl^−^ (mmol/L)	105.32, 4.80	103.25, 7.09	104.45, 5.94	0.059
Serum Creatinine (μmol/L)	94.73, 88.94	121.89, 76.66	106.20, 84.75	0.066
BNP (pg/mL)	97.14, 142.51	295.08, 308.15	187.27, 252.10	**<0.001**
Troponin (ng/mL)	0.02, 0.08	0.14, 0.67	0.07, 0.44	0.190
**Portal vein doppler ultrasound findings**				
The diameter of the main portal vein (mm)	11.54, 2.40	11.43, 2.89	11.49, 2.59	0.826
The blood flow velocity of the portal vein (cm/s)	16.42, 4.46	17.32, 5.10	16.77, 4.72	0.324
The inner diameter of the main splenic vein (mm)	8.15, 2.34	8.02, 2.70	8.10, 2.48	0.804
The blood flow velocity of the spleen (cm/s)	18.45, 7.15	20.95, 5.03	19.44, 6.48	0.067

*The bold values of “P” represent statistical differences between control and atrial arrhythmia group*.

### Complications and Child-Pugh Score of Liver Cirrhosis Patients With or Without Atrial Arrhythmia

The complications of liver cirrhosis (ascites, hepatic encephalopathy, and gastroesophageal varices) are presented in Table 4. Among 135 liver cirrhosis patients, 53 (60.74%) had ascites and 4 (2.96%) had hepatic encephalopathy. Additionally, 33 patients (24.44%) had no gastroesophageal varices, 89 patients (65.93%) had gastroesophageal varices but no variceal bleeding, and 13 patients (9.63%) had gastroesophageal varices and variceal bleeding. The results of the Chi-square test suggested that liver cirrhosis patients with ascites were more likely to present with atrial arrhythmia (χ^2^ = 31.076, *P* < 0.001), but there were no significant differences related to hepatic encephalopathy or gastroesophageal varices between the two groups.

**Table 4:** The complication of liver cirrhosis patients with or without atrial arrhythmia.

**Complication**	**Control**	**Atrial arrhythmia**	**Total**	**χ^2^**	***P***
	**(*n* = 78)**	**(*n* = 57)**	**(*n* = 135)**		
**Ascites**					
No	63	19	82 (60.74%)	31.076	**<0.001**
Ascites	15	38	53 (39.26%)		
**Hepatic encephalopathy**					
No	74	50	124 (91.85%)	2.251	0.202
Hepatic encephalopathy	2	2	4 (2.96%)		
**Gastroesophageal varices**					
No	13	20	33 (24.44%)	9.949	0.271
No variceal bleeding	60	29	89 (65.93%)		
Variceal bleeding	5	8	13 (9.63%)		

*The bold values of “P” represent statistical differences between control and atrial arrhythmia group*.

The Child-Pugh score [calculated on the basis of prolonged PT (PT: 9.8–14.5 s), serum levels of total bilirubin and ALB, severity of ascites, and hepatic encephalopathy] of liver cirrhosis patients with or without atrial arrhythmia are presented in Table 5. The results showed that the Child-Pugh scores of liver cirrhosis patients with atrial arrhythmia (8.07 ± 2.22) were significantly higher than those of liver cirrhosis patients without atrial arrhythmia (7.03 ± 1.84) (*P* = 0.003).

**Table 5:** The Child-pugh score of liver cirrhosis patients with or without atrial arrhythmia.

**Liver function classification**	**Control**	**Atrial arrhythmia**	**Total**	***P***
	**(*n* = 78)**	**(*n* = 57)**	**(*n* = 135)**	
Child-pugh score	7.03 ± 1.84	8.07 ± 2.22	7.49 ± 2.09	**0.003**

*The bold values of “P” represent statistical differences between control and atrial arrhythmia group*.

### Multivariate Logistic Regression Analysis of Factors Predicting Atrial Arrhythmia in Liver Cirrhosis Patients

In this study, multivariate logistic regression analysis was used to identify the risk factors for atrial arrhythmia in patients with liver cirrhosis. The results of multivariate logistic regression analysis of significantly different factors (age, ALB, serum Na^+^, BNP, ascites, and Child-Pugh score) are presented in Table 6. The results suggested that age (β = 0.094, OR = 1.098, 95% CI 1.039–1.161, *P* = 0.001) and ascites (β = 1.354, OR = 3.874, 95% CI 1.202–12.483, *P* = 0.023) were significantly associated with atrial arrhythmia.

**Table 6:** Multivariate logistic regression analysis of factors associated to atrial arrhythmia in liver cirrhosis patients.

**Variable**	**β**	**Odds ratio**	**95% CI**		***P***
			**Lower**	**Upper**	
Age	0.094	1.098	1.039	1.161	**0.001**
WBC (×10^9^/L)	0.080	1.084	0.866	1.356	0.482
ALB (g/L)	0.026	1.026	0.902	1.168	0.692
Serum Na^+^ (mmol/L)	−0.028	0.972	0.879	1.075	0.582
BNP (pg/mL)	0.004	1.004	1.000	1.008	0.063
Child-Pugh score	0.043	1.044	0.689	1.581	0.839
Ascites	1.354	3.874	1.202	12.483	**0.023**

*The bold values of “P” represent statistical differences between control and atrial arrhythmia group*.

## Discussion

In the present study, we aimed to identify the risk factors associated with new-onset atrial arrhythmia in patients with cirrhosis. We enrolled 135 patients with liver cirrhosis and performed a retrospective study. Seven clinical variables were found to be significantly different between liver cirrhosis patients with or without arrhythmia. These factors were age, WBC, ALB, serum Na^+^, BNP, ascites, and Child-Pugh score. Once multivariate logistic regression analysis and multivariable adjustment were performed, we found that age and ascites were two risk factors associated with atrial arrhythmia in patients with liver cirrhosis.

In the past few decades, the number of liver cirrhosis-related deaths has steadily increased (20, 21). Globally, significant medical resources are devoted to the treatment and care of patients with liver cirrhosis every year (22–25). Previous studies have demonstrated that liver cirrhosis may cause cirrhotic cardiomyopathy, which is related to atrial arrhythmia, and particularly atrial fibrillation (7, 26).

Our data suggest that patients with liver cirrhosis with atrial arrhythmia are about 10 years older than those without atrial arrhythmia. Multivariate logistic regression analysis showed that atrial arrhythmia was significantly associated with advanced age in our study. Similarly, Mwalitsa and Gundling found that the occurrence of atrial fibrillation is positively correlated with age (27, 28). Previous studies confirmed that atrial arrhythmia is more likely in elderly patients, and the prevalence of atrial fibrillation is associated with increasing age (29). Additionally, our data suggested that liver cirrhosis patients with ascites were more likely to develop atrial arrhythmia. In general, myocardial diastolic dysfunction occurs before systolic dysfunction in patients with liver cirrhosis and ascites (30). Myocardial diastolic dysfunction is mainly due to myocardial hypertrophy and fibrosis, and consequently induces structural heart disease and arrhythmia (31–34). We hypothesized that ascites induced myocardial diastolic dysfunction due to disorders of peripheral blood circulation, which may be a possible mechanism by which atrial arrhythmia develops. However, a direct correlation between ascites and atrial arrhythmia has not been reported previously. In the present study, we thus identified a novel risk factor for atrial arrhythmia in patients with liver cirrhosis.

In the present study, there were significant differences in ALB, BNP, serum Na^+^, and Child-Pugh scores between liver cirrhosis patients with atrial arrhythmia and those without atrial arrhythmia. However, after correction, these factors did not correlate with arrhythmia. Previous studies have been controversial regarding the correlation between the severity of liver disease and cardiac dysfunction (27, 31). In theory, it is considered that the severity of liver cirrhosis correlates with an increased risk of atrial arrhythmia due to the severe complications associated with higher Child-Pugh scores, such as hypoalbuminemia and the disturbance of water and electrolyte balance (29, 35). However, our data suggest that there is no correlation between Child-Pugh scores and the occurrence of atrial arrhythmia in patients with liver cirrhosis. Our data showed that ALB and serum Na^+^ levels were significantly decreased in liver patients with atrial arrhythmia, which may be associated with the development of ascites. In patients with liver cirrhosis, the decrease in serum Na^+^ caused by water and sodium storage, as well as the inversion of the A/G ratio and the subsequent decrease of plasma colloidal osmotic pressure caused by insufficient synthesis of ALB, are all essential factors in the development of ascites (36, 37). A prospective clinical study found that BNP, the most sensitive biochemical marker, is associated with the MELD score in Child-Pugh C patients (38). It has been reported that BNP is a predictive factor of cardiac decompensation risk in patients with liver cirrhosis after TIPS (39). In the present study, there was no correlation between the significant increase in BNP and the occurrence of atrial arrhythmia, which was probably due to the inclusion of Child-Pugh C patients and Child-Pugh A and B patients.

The occurrence of atrial arrhythmia in patients with liver cirrhosis may be related to the following reasons. In liver cirrhosis, bile acid metabolism disorder may promote the occurrence of atrial arrhythmia (40). In addition, when liver cirrhosis patients are accompanied by ascites, a hyperdynamic circulatory state due to simultaneous splanchnic and peripheral arterial vasodilatation leads to changes in autonomy, excitability, and conductivity of cardiomyocytes (41). The dysfunction of the autonomic nervous system also plays an important role in the occurrence of atrial arrhythmia.

## Limitations

The present study has several limitations. First, the retrospective study had some unavoidable limitations, such as selection bias, which inevitably affect the results (42). Second, the 135 liver cirrhosis patients enrolled were all from a single center, the small sample size was small, and limited clinical information was available. Third, there may be significant differences in factors that were not measured in the present study. As data were collected retrospectively, and patient treatment varied according to individual differences, some clinical information was lacking, particularly the results of portal vein Doppler ultrasound investigations. Related research that draws on data from multiple centers is needed in future studies in order to continue to establish the risk factors for atrial arrhythmia in patients with liver cirrhosis.

## Conclusion

In conclusion, the present study identified age and ascites as two risk factors associated with atrial arrhythmia in patients with liver cirrhosis. Among patients with liver cirrhosis, elderly patients and patients with ascites are more likely to develop atrial arrhythmia. Patients with liver cirrhosis should undergo regular ECG examinations to detect atrial arrhythmia and active management of both liver cirrhosis and atrial arrhythmia should be practiced, particularly in elderly patients and in patients with complicated ascites.

## Data Availability Statement

The raw data supporting the conclusions of this article will be made available by the authors, without undue reservation.

## Ethics Statement

The studies involving human participants were reviewed and approved by Ethics Committee of Tongji Hospital Affiliated to Tongji University. The patients/participants provided their written informed consent to participate in this study. Written informed consent was obtained from the individual(s) for the publication of any potentially identifiable images or data included in this article.

## Author Contributions

XL collected the clinical information of the enrolled patients, analyzed the data, and drafted the manuscript. ZW and LY collected the clinical information of the enrolled patients. CY and MS supervised the research design and revised the manuscript. All authors contributed to the article and approved the submitted version.

## Conflict of Interest

The authors declare that the research was conducted in the absence of any commercial or financial relationships that could be construed as a potential conflict of interest.
